# DIPG in Children – What Can We Learn from the Past?

**DOI:** 10.3389/fonc.2015.00237

**Published:** 2015-10-21

**Authors:** Magimairajan Issai Vanan, David D. Eisenstat

**Affiliations:** ^1^Department of Pediatrics and Child Health, University of Manitoba, Winnipeg, MB, Canada; ^2^Department of Biochemistry and Medical Genetics, University of Manitoba, Winnipeg, MB, Canada; ^3^Department of Pediatrics, University of Alberta, Edmonton, AB, Canada; ^4^Department of Medical Genetics, University of Alberta, Edmonton, AB, Canada; ^5^Department of Oncology, University of Alberta, Edmonton, AB, Canada

**Keywords:** diffuse intrinsic pontine glioma, blood–brain barrier, pediatric neuro-oncology, brain tumor, re-irradiation

## Abstract

Brainstem tumors represent 10–15% of pediatric central nervous system tumors and diffuse intrinsic pontine glioma (DIPG) is the most common brainstem tumor of childhood. DIPG is almost uniformly fatal and is the leading cause of brain tumor-related death in children. To date, radiation therapy (RT) is the only form of treatment that offers a transient benefit in DIPG. Chemotherapeutic strategies including multi-agent neoadjuvant chemotherapy, concurrent chemotherapy with RT, and adjuvant chemotherapy have not provided any survival advantage. To overcome the restrictive ability of the intact blood–brain barrier (BBB) in DIPG, several alternative drug delivery strategies have been proposed but have met with minimal success. Targeted therapies either alone or in combination with RT have also not improved survival. Five decades of unsuccessful therapies coupled with recent advances in the genetics and biology of DIPG have taught us several important lessons (1). DIPG is a heterogeneous group of tumors that are biologically distinct from other pediatric and adult high grade gliomas (HGG). Adapting chemotherapy and targeted therapies that are used in pediatric or adult HGG for the treatment of DIPG should be abandoned (2). Biopsy of DIPG is relatively safe and informative and should be considered in the context of multicenter clinical trials (3). DIPG probably represents a whole brain disease so regular neuraxis imaging is important at diagnosis and during therapy (4). BBB permeability is of major concern in DIPG and overcoming this barrier may ensure that drugs reach the tumor (5). Recent development of DIPG tumor models should help us accurately identify and validate therapeutic targets and small molecule inhibitors in the treatment of this deadly tumor.

## Introduction

The anatomic brainstem includes three distinct regions: the midbrain, pons, and medulla oblongata. Tumors of the brainstem constitute approximately 10–15% of all central nervous system (CNS) tumors in children ages 0–14 years in the USA; the majority of these tumors are of glial origin (gliomas) (1). Prior to modern imaging techniques, all brainstem gliomas were regarded as a single pathological entity, and the prognosis was considered uniformly poor. Computerized tomography (CT) and Magnetic Resonance Imaging (MRI) have been used to classify brainstem tumors based on their growth pattern and amenability to surgical resection. Diffuse intrinsic pontine gliomas (DIPG) are the most common brainstem tumors in children and account for greater than 80% of brainstem gliomas in this age group. The widespread infiltrative nature of this tumor, predominantly high-grade features, coupled with its critical anatomic location and inoperability, has led to a uniformly poor outcome in children with DIPG.

### Clinical Features/Diagnosis

DIPG occurs in all age groups but is most commonly diagnosed in children between the ages of 5 and 10 years. There is equal predilection for both sexes (M:F = 1:1), and the prognosis is significantly worse than that of other brainstem tumors. Due to local infiltration and brainstem localization, DIPGs are not amenable to surgical resection. The clinical features of patients with brainstem tumors vary with respect to tumor location and the nature/pattern of growth of these tumors. Patients with DIPG have a short latency (usually <2–3 months) between the onset of clinical symptoms and diagnosis. The classic triad of symptoms includes *cerebellar signs* (e.g., ataxia, dysmetria, dysarthria), *long*-*tract signs* (e.g., increased tone, hyperreflexia, clonus, Babinski sign, motor deficit, etc.), and isolated or multiple *cranial nerve palsies* (unilateral or bilateral), more commonly sixth and seventh cranial nerve palsies. Patients with diffuse brainstem tumors associated with neurofibromatosis type 1 (NF1) may mimic DIPG on imaging. However, in the context of NF1, these are usually low-grade gliomas (LGG, WHO grades I–II) that can be asymptomatic or diagnosed in the context of an insidious history of isolated cranial nerve palsy or motor deficit. Careful clinical examination for the stigmata of NF-1 and family history should help in the identification of these lesions that usually do not require any active treatment. In DIPG, signs and symptoms of increased intracranial pressure (due to obstructive hydrocephalus from expansion of the pons) are seen in <10% of children. Various other non-specific symptoms present either at the time of diagnosis or at the time of progression include sensory abnormalities, behavioral changes, urinary problems, declining school performance, and respiratory symptoms including sleep apneas.

Over the years, many classification schemes have been proposed for brainstem tumors, and most have utilized the best neuroimaging modalities available at the time of classification (2). The earliest classifications relied on CT and surgical observations; however, the more recent schemes include MRI. In the broadest sense, these tumors are divided into two groups, either focal or diffuse. The more complex schemes subdivide these tumors by location within the brainstem (midbrain, pons, or medulla), growth pattern (intrinsic or exophytic), direction and extent of tumor growth, the presence or absence of contrast enhancement, and the presence of hemorrhage or hydrocephalus (3). In one of the most recent classifications proposed by Choux et al. based on both CT and MRI characteristics, brainstem tumors are divided into four types (4). DIPGs are classified as type I tumors (not to be confused with WHO grade I). These lesions appear hypointense on CT with non-delineated borders and do not significantly enhance on T1-weighted MRI sequences with gadolinium as the contrast reagent. DIPGs are characterized by diffuse infiltration and swelling of the brainstem.

### Why will Biopsy of DIPG Become Important?

Historically, in the pre-CT and MRI eras, stereotactic brainstem biopsies were performed on a routine basis for histological diagnosis of DIPG. Due to the heterogeneity of these tumors, the significant morbidity potentially associated with the biopsies, the limited therapeutic options available based on biopsy results, the prevalence of poor candidates for biopsy at the time of presentation (i.e., those with focal neurological deficits, increased ICP), and the widespread availability of MRI with characteristic imaging findings, routine biopsy as the standard of care was discontinued in the early 1990s (5).

### Safety and Feasibility of the Procedure

Many centers in Europe have been performing routine diagnostic biopsies of children with suspected DIPG. In two of the largest series of brainstem tumor biopsies in children (6, 7), there were only transient reversible morbidities (in <10% of patients) and no mortality reported. In a retrospective analysis, Cartmill et al. (6) reviewed 18 brainstem glioma patients who underwent CT-guided stereotactic biopsy. All 18 patients had a histological diagnosis of glioma [HGG, *n* = 13 (glioblastoma multiforme, GBM = 8; anaplastic astrocytoma (AA), *n* = 5) and low grade gliomas, *n* = 5]. Besides the five patients who developed a transient increase in their neurological deficits post-operatively (hemiparesis, increased eye movements, and VII nerve palsy), there was no mortality reported. Roujeau et al. (7) reported a prospective series of 24 patients with DIPG who underwent stereotactic biopsy. In two patients, a diagnosis of low-grade gliomas was made (LGG and pilocytic astrocytoma) and each was followed with an initial period of observation before initiating radiotherapy. Only two patients had transient post-operative neurological deficits (cranial nerve palsy and exacerbation of pre-operative hemiparesis) and no deaths were reported. A historical cohort study by Cage et al. (8) of nine patients with DIPG who underwent stereotactic biopsy revealed no intra-operative complications and only one patient with post-operative neurological deficit (seizures with hydrocephalus), not directly related to the procedure. In their review of literature, Cage et al. (8) report >300 pediatric cases of DIPG who underwent biopsy with mortality attributable to the procedure in only two cases. In a recent retrospective series from Wang et al., only 3 of the 15 cases of DIPG who underwent biopsy had transient new or worsening neurological deficits and there was no biopsy related mortality (9). Careful pre-operative planning using functional imaging like Diffusion Tensor Imaging tractography to delineate the white matter (especially corticospinal tracts) and PET imaging to identify regions of interest will further decrease the morbidity and increase the diagnostic yield of the biopsy (10, 11). The post-operative morbidity of image guided stereotactic biopsy of intrinsic pontine lesions is comparable to biopsy procedures in other brain locations.

### Sampling Bias, Diagnostic Yield and Differential Diagnosis

In all of the biopsy series of DIPG cases (6–9), the pathologic diagnosis revealed low grade as well as HGG. Initial concerns regarding the heterogeneity of the tumor leading to sampling bias and downgrading of the tumor (from WHO grade IV to grade II) resulting in less intense therapy, may no longer be considered valid. Recent data from a large series of patients suggests that there are distinct histologic subgroups of DIPG with varying clinical and molecular features (12). Although the patients may present with typical clinical features of DIPG, the histopathology is diverse (spectrum from grade II to IV) and low grade tumors can behave clinically as aggressively as HGGs (8, 12). There is potential intra-tumor histologic heterogeneity seen in DIPG as demonstrated in the series by Buczkowicz et al. (12). Tumors with classic GBM histology show grade II or grade III histology in some areas, which may be selected by biopsy. Furthermore, these heterogeneous tumors had classic GBM histologic features in the pons but demonstrated low grade astrocytoma (II) or AA (III) features in the thalamus or brainstem. These observations suggest that histologic grading using current WHO criteria may not be appropriate for diagnostic purposes in DIPG. The diagnostic yield of biopsy specimens in DIPG is very high (90–100%) (13). Although the amount of tumor tissue obtained is limited by the needle size, it is usually sufficient to make a histopathologic diagnosis. Moreover, recently described biopsy techniques permit multiple biopsies under one anesthetic, thereby providing sufficient tissue not only for diagnosis but also for molecular studies and primary cell culture (8, 13). Apart from gliomas (grade II–IV), Primitive Neuro-ectodermal Tumors (PNET) (9, 10, 12) and rarely germ cell tumors (including germinomas and teratomas) can present as diffuse infiltrative brainstem tumors; the management of these tumor entities is different than for gliomas. Non-malignant conditions presenting as brainstem lesions include neurodegenerative conditions (Alexander disease, acute demyelinating encephalomyelitis, central pontine myelinolysis) and infections (such as paracoccidioidomycosis) (14).

### Areas of Controversy

Some neurosurgeons and other clinicians have introduced the terms typical and atypical DIPG and advocate for biopsy for atypical disease presentations. Literature review on pediatric brainstem gliomas suggests the following criteria for a *typical* DIPG: a combination of *clinical symptoms and signs with short latency *<*3–6 months* – triad of: (1) cerebellar signs, e.g., ataxia, dysmetria and dysarthria; (2) long tract signs: hypertonia, hyper-reflexia, motor deficits; and (3) cranial nerve palsies-isolated or multiple sixth, seventh) and *radiological findings on MRI*: (1) an intrinsic, centrally located tumor involving greater than 50–66% of the axial diameter of the pons with (2) hypointensity on T1 images, (3) hyperintensity on T2 images with (4) indistinct tumor margins with engulfment of basilar artery and (5) no cystic or exophytic components (15–17). However, a survey conducted among neurosurgeons revealed that there was no consensus regarding what constitutes a typical DIPG on MRI (18). The *differential diagnosis* for a brain stem tumor in a child with unusual presentation and atypical findings on neuroimaging include embryonal tumors like ATRT, PNET, and non-malignant lesions like infections, neurodegenerative conditions and hemangioblastomas (9, 10, 12, 14); the management of these conditions is entirely different than for gliomas. Focal brainstem tumors usually have insidious onset, prolonged latency and atypical clinical features like failure to thrive, emesis etc. Hence, it may be reasonable for biopsy to be considered in those patients with atypical features for whom clinical management decisions would change when provided with a diagnosis other than DIPG.

In a child with typical imaging appearance of DIPG, the current recommendations are to offer upfront biopsy only in a clinical trial setting (19).

The ability to identify the whole mutational landscape of DIPG using tissue obtained by small-needle pretreatment biopsies (20) coupled with availability of antibodies to detect (a) K27M mutations in both Histone 3 (H3) variants (H3.3 and H3.1) (21, 22) and (b) loss of tri-methylation (anti-HEK27me3) due to these mutations provides opportunities to improve diagnosis, assignment of prognosis, and identification of potentially druggable targets (PDGFRA, ACVR1, and FGFR1) (12). There is sufficient evidence in the literature to suggest that as the field moves forward, testing for H3K27M mutations will help in diagnosis (12, 23), stratification into subgroups (12, 24) and prognosis (12, 23) and will be included in the design of prospective clinical trials testing new agents and treatment approaches. In the future, clinical and molecular subgroup-based patient stratification in multi-center clinical trials of DIPG will include stereotactic biopsy when it is considered safe to proceed in individual patients.

## Is DIPG a Whole Brain Disease?

The concept of focal radiotherapy of DIPG is based in part by reports suggesting that the tumor mostly spreads by contiguity and the recurrence is mostly local, within the radiotherapy fields (25–27). Since distant failure is not considered a common phenomenon in the natural history of DIPG, patients do not routinely undergo neuraxis imaging either at diagnosis or follow up after initial therapy. Leptomeningeal dissemination/disease (LMD) was reported in 4–33% of patients in the pre-MRI era (25–27). In contrast, several studies have identified widespread CNS disease by autopsy and/or neuroimaging (12, 28–35). In fact, disseminated disease may be seen (1) at the time of diagnosis; (2) during follow-up while on treatment; or (3) at the time of autopsy. Benesch et al. (30) in their retrospective data analysis of HGGs report an incidence of 3.1% (*N* = 10/546, HGG = 348, DIPG = 198) of patients with primary dissemination at diagnosis. However, there was only one case of DIPG (*n* = 1/198, 0.5%) with distant spread (lateral ventricle). Sethi et al. (33) reported 3/9 patients (33%) who had LMD detected at diagnosis. In the same series, prospective neuraxis surveillance (MRI of brain and spine) at 4-month intervals after completion of therapy revealed local failure in 75% of patients (12/15) and LMD in six patients (6/9, 66%). Moreover, patients with LMD had shorter overall survival (OS) when compared to patients with localized disease: 12.0 ± 3.3 months without LMD and 8.0 ± 2.1 months with LMD (*P* = 0.059). Donahue et al. (28) reported 15/18 (83%) children with DIPG with progression of disease of which 14 had local failures. The protocol on which these children were treated mandated monitoring with neuraxis surveillance MRIs every 4 months after completion of treatment. Interestingly, none of these patients had clinical evidence of LMD. The authors concluded that LMD occurs primarily in the setting of local progression and therapy should be directed primarily toward improving local control.

Wagner et al. (31) reported secondary disseminating disease (SDD) in DIPG in 13% of their patients (14/110). The median time between diagnosis and SDD was 7.2 months (range, 4.6 months to 2.2 years) and there was parenchymal metastasis involving the supratentorial, infratentorial, and spinal regions along with LMD. DIPG patients who had LMD and ventricular tumor spread had decreased OS when compared to patients who had parenchymal metastasis. However, SDD did not reduce OS in DIPG tumors, suggesting that rapid local progression of disease is probably the main cause of shortened survival. Gururangan et al. (32) in a retrospective review of neuraxis dissemination in DIPG found metastatic disease in 17.3% (18/96) patients. All the patients died of disease at a median of 5 months (range, 0.5–20) from the diagnosis of neuraxis spread. The pattern of spread showed both LMD and parenchymal involvement. Yoshimura et al. (29) in their autopsy series of DIPG reported more than 50% of patients had disseminated disease with supratentorial spread in 45% (18/40) and LMD in 32.5% (13/40). Almost all the cases except one were HGGs (GBM = 34, AA = 5). Caretti et al. (35) in their autopsy series of 16 DIPG patients found widespread extension involving the midbrain/medulla (63%), cerebellum (56%), thalamus (56%), frontal cortex (25%), and supratentorial leptomeninges (25%). There was disease spread into the spinal cord in two out of the three patients examined. A unique form of sub-ventricular spread resulting in tumor nodules seen in the frontal horns of the lateral ventricles was observed in 10 of 16 cases (62.5%). In one of the largest series of DIPG (12), 38% of patients exhibited LMD of their tumor. There was parenchymal spread in the form of diffuse involvement of brainstem, spinal cord and supratentorial (thalamus and frontal lobe) regions. There was no association between histology and LMD. However, there were TP53 mutations in 6 of the 11 patients tested and none of the patients with LMD were found to have the ALT (alternate lengthening of telomeres) phenotype. Histone H3 K27M mutations in H3.3 were seen in 67% (35/52) and in H3.1 were seen in 15% (8/52). Histone H3 mutations were observed in all histology grades of DIPG (II–IV): 88% in GBM, 60% of AA, and 71% of low grade astrocytomas. K27M-H3.3 mutations were higher in GBM patients (78%) when compared to AA histology (33%) (*P* = 0.0016). Importantly, low grade tumors carrying H3.3 mutations had OS similar to that of GBM tumors and patients with high grade tumors with wild-type H3.3 had OS comparable to wild-type low grade astrocytomas (12).

### Diagnostic and Therapeutic Implications

At the time of diagnosis, whenever available, biopsy specimens of DIPG are suggested to be tested for Histone K27M mutations (H3.3 and 3.1) along with routine WHO histological grading and other markers (TP53, PDGFRA, ACVR1, and FGFR1). This will help in obtaining the correct diagnosis, proper patient selection for clinical trials (for risk stratification and targeted therapies) and prognosis (12, 36). Neuroimaging at the time of diagnosis should include MRI of the brain; the spine may be included at the discretion of the attending physician. Thereafter, regular interval neuraxis imaging during follow-up could be included in prospective clinical trials to better understand the natural history of DIPG progression, including disease dissemination. This data may help guide therapy decisions and prognosis.

The majority of DIPG patients treated with focal brainstem radiotherapy (RT) relapse locally. There are several reports, which suggest that sub-lethal irradiation (IR) promotes migration of glioma cells (37–40). Wild-Bode et al. (37) proposed that tumor cells stimulated to invade by sub-lethal doses of irradiation may escape the target volume of post-operative RT, thereby evading delivery of a cumulative lethal dose and forming the basis for loco-regional relapse a few months after RT. These infiltrative tumors are less proliferative as shown by few mitoses and scarce MIB1 immunopositivity (12). Therefore, concurrent anti-invasive/anti-migratory therapies (cell cycle/check point inhibitors, glycogen synthase kinase-3 inhibitors: lithium chloride and 6-bromoindirubin-oxime) along with RT have been suggested to be beneficial in these patients potentially improving the efficacy of RT (40, 41).

The question of whether to offer focal vs. cranio-spinal RT in DIPG cannot be currently answered due to the paucity of data. Benesch et al. (30) in their series of HGG (including one patient with DIPG) did not observe a statistically significant difference in the progression free survival (PFS) and OS between patients with and without tumor dissemination at diagnosis. Hence, their recommendation was to treat both the groups in the same manner (focal IMRT). Similarly, data from the German registry (42) comparing six HGG patients who had metastasis at diagnosis (two patients with DIPG) and underwent intensified chemo-radiotherapy (CSI ± local boost to tumor deposits with concurrent metronomic temozolomide) demonstrated inferior results when compared to the Benesch series (30). In the future, a multi-centered randomized clinical trial to address the issue of appropriate clinical target volume (CSI vs. focal RT) in DIPG may be considered. Although recurrence of tumor (either locally or at distant sites) has shorter OS, CNS prophylaxis in the form of regular intra-thecal chemotherapy (43) similar to acute lymphoblastic leukemia (ALL) therapy is not recommended.

## Treatment

Diffuse intrinsic pontine glioma is almost invariably fatal with a mean OS of 8–14 months from the time of diagnosis. Radiation therapy (RT) prolongs survival by a mean of 3–6 months, but is still considered aggressive palliative therapy. A number of adjuvant therapies such as chemotherapy, targeted therapies, differentiation agents and radiation sensitizers have been studied, but none of them have had any significant impact on patient outcomes. There is no role for surgery in a typical case of DIPG. However, recent advances in understanding the biology of these tumors and availability of targeted therapies may warrant biopsy at diagnosis in some patients in order to personalize therapy and/or enroll the child in a clinical trial.

### Radiation Therapy

The current standard of care for newly diagnosed DIPG patients is fractionated focal intensity modulated radiation therapy (IMRT) to the tumor along with 1–2 cm margins (54–60 Gy, 1.8–2 Gy fractions, given once daily for 5 days per week over a period of 6 weeks). Supportive care in the form of corticosteroids is used to treat the peri-tumoral edema and is usually weaned after completion of RT. About 70–80% of patients respond to RT with some transient improvement in their neurological symptoms but more than half of the patients incur steroid induced side effects. Lower doses of RT (<50Gy) have worse outcomes, and higher doses using hyper-fractionated RT (66–78 Gy) do not appear to provide a survival advantage when compared to standard dose and fractionation protocols (44–54) (Table 1). It was postulated that re-population by rapidly proliferating tumor cells may be a reason for local treatment failure and accelerated fractionation (conventional fraction sizes twice daily) was expected to reduce the potential for re-population and improve outcomes. Conversely, several studies using hypo-fractionation have reported results that are similar to those observed with normal fractionation but offer no distinct survival benefits (52–54). Although the advantage of the hypofractionation technique is reduction in the total duration of treatment (13–18 fractions instead of 30–33), the major disadvantages are the adverse effects of RT on adjacent normal tissue. Hyperfractionation usually requires twice daily anesthetics in young children. Following completion of radiation, there is clinical progression of disease in almost all cases with radiologic evidence of local recurrence within 3–6 months. As discussed above, there could be LMD as well as distant parenchymal recurrence. Although RT appears beneficial, radiation sensitizers have not shown any improvement in outcomes to date. The use of RT concurrently with radio-sensitizers such as platinum compounds (carboplatin), topoisomerase inhibitors (etoposide, trofosfamide, topotecan), and other agents (metronomic temozolomide, etanidazole) has not been successful (Tables 1 and 3A,B).

**Table 1:** **Radiation therapy in DIPG**.

Reference	Year/phase	Number of patients (*N*)	Treatment: total dose (Gy)/total fractions/dose per fraction (Gy)	Imaging	Outcome	Comments
Jenkin et al. (44)	1987/RCT	33	50–60/–/0.8–0.9	CT	OS: 20%	Patients randomized to RT with and without adjuvant chemotherapy: CCNU, vincristine and prednisone Procarbazine was given at relapse in patients who received chemotherapy only Brain stem gliomas (including DIPG) were included in the study
Packer et al. (45)	1987/pilot	16	64.8/54/1.2	CT/MRI	Median PFS: 7 months	
Freeman et al. (46)	1988/I–II	34	66/60/1.1	CT	MST: 11 months OS at 1 year: 48%	Brain stem gliomas (including DIPG) were included in the study
Edwards et al. (47)	1989/I–II	34	72/72/1	CT/MRI	MST: 16 months Median TTP: 11 months	Brain stem gliomas (including DIPG) were included in the study
Freeman et al. (48)	1991/I–II	57	70.2/60/1.17	CT/MRI	Median TTP: 6 months. MST: 10 months OS at 1 year – 39.6 ± 6.6%, at 2 years – 23 ± 5.8%	
Packer et al. (49)	1993/I–II	53	72/72/1	CT/MRI	OS at 1 year – 38 ± 6.5%, at 2 years – 14 ± 5.4%, at 3 years – 8 ± 6.5	
Freeman CR et al. (27)	1993/I–II	39	75.6/60/1.26	MRI	Median TTP: 7 months. MST: 10 months OS at 1 year – 39.9 ± 8.3%, at 2 years – 7 ± 4.8%	
Packer et al. (50)	1994/I–II	66	78/78/1	CT/MRI	OS at 1 year – 35 ± 6%, at 3 years – 11 ± 6%	
Packer et al. (51)	1996/pilot	32	72/72/1	MRI	Median TTP: 5 months	Recombinant beta-interferon was used prior to and with RT Brain stem gliomas (including DIPG) were included in the study
Lewis et al. (52)	1997/pilot	28	48.6/27/1.8 50.4/28/1.8	CT/MRI	MST: 8.5 months OS at 1 year – 32%, at 2 years – 11%	Brain stem gliomas (including DIPG) were included in the study Biopsy was done in 10 cases with Grade I/II (*n* = 4) and Grade-III/IV (*n* = 6)
Janssens et al. (53)	2009/pilot	9	39/9/3 (*n* = 8) 33/6/5.5 (*n* = 1)	MRI	Median TTP: 4.9 months OS: 8.6 months	Biopsy proven Grade IV astrocytomas in four patients
Negretti et al. (54)	2011/–	22	49/15/3	MRI	Median TTP: 5.7 months OS: 7.6 months	

*CT, computerized tomography; MRI, magnetic resonance imaging; OS, overall survival; PFS, progression free survival; MST, median survival time; TTP, time to tumor progression*.

#### Feasibility of Palliative Re-Irradiation in DIPG

There is evidence suggesting that re-irradiation in pediatric brain tumors (posterior fossa ependymoma, EP) is well tolerated and provides durable local tumor control (55, 56). Median time from first to second irradiation was 21.9 months (range, 7.5–67.7 months) (55) in one series and 26 months (range, 13–88 months) in another series (56). The median re-RT dose for focal fractionated re-irradiation (FFRT) was 55.2 Gy (range, 50.4–54 Gy) with a cumulative dose of 87–113 Gy to the brain stem and none of the patients had radiation necrosis (55).

Based on these studies in ependymoma and in the absence of effective treatment options for recurrent DIPG, some institutions are providing palliative re-irradiation with/without concurrent chemotherapy for DIPG (57, 58). Fontanilla et al. (57) treated six recurrent DIPG patients (clinical and MRI confirmation) with chemo-radiotherapy followed by post RT chemotherapy. The time to initial progression from the completion of first RT was 4 to 18 months and the time interval between initial RT and re-RT was 8–28 months. The re-RT dose was between 18 Gy (*n* = 1) to 20 Gy (*n* = 4). There was good symptomatic response in four of the six patients with improvement in speech, swallowing and ambulation. The median clinical progression-free survival (PFS) time was 5 months and there were minimal RT related side effects with no Grade 3–4 toxicities reported. Massimino et al. (58) re-irradiated 11 of their 16 locally relapsed DIPG patients. There was tumor volume shrinkage in 7 cases and steroids were weaned completely in 10 cases. The median survival after re-RT was 6 months (range, 6 weeks to 14 months) and none of the re-irradiated patients showed any evidence of worsening symptoms or unexpected side effects. Preferred candidates for re-irradiation in DIPG may include those children who have experienced durable responses to prior radiation without evidence of clinical/radiological progression off therapy (57, 59).

We analyzed data on 10 patients of recurrent DIPG treated with re-RT across different centers in Canada (Table 2; 60). Median time from diagnosis to progression was 12 months (range, 4–37 months) in this cohort, exceeding median time to progression in historical controls. Re-irradiation total dose varied between institutions from 21.6 Gy (*n* = 2), 30.6 Gy (*n* = 6), and 36 Gy (*n* = 2), administered in 1.8 Gy daily fractions. Re-radiation was to the involved field except in two patients who received whole brain irradiation due to distant/disseminated relapse. One patient received a third course of focal radiation (21.6 Gy) 6 months after re-radiation. Re-irradiation was tolerated very well by all patients. Four patients had transient fatigue and decreased appetite during treatment. All but one showed neurological improvement, with four patients showing full recovery. With a median follow-up from diagnosis of 19.5 months (range, 9–45 months), seven patients died, with a median time from re-irradiation to death of 9 months (range, 5–13 months). When compared to an historic cohort of 46 patients, median time from progression to death was 91.5 days in the non re-irradiated patients, vs. 171 days in the re-irradiated ones (*P* < 0.05). Although these preliminary results are encouraging, they are from small pilot studies and remain to be confirmed in a clinical trial setting. Although we do not suggest that re-irradiation should become the standard of care, based upon published and unpublished results, we encourage the development of prospective cooperative group Phase II studies offering re-irradiation so that this treatment option can be properly studied from a safety and efficacy perspective. Based on the above data, a dose range from 30 to 36 Gy in 16 to 20 fractions (1.8 Gy per fraction) at the time of recurrence may be a reasonable approach that is feasible and well tolerated.

**Table 2:** **Re-irradiation for DIPG (60)**.

Patient/sex	Age at Dx	Time from Dx to Relapse (m)	Time from Relapse to start re-RT (d)	Dose of re-RT (Gy)	MRI post re-RT	DXM	Clinical (neurologic symptoms) improvement after re-RT	Side effects after re-RT	Time from re-RT to progression (m)	Outcome/follow up (m)
1/F	6 years 5 months^a^	14	20	30.6/focal	Yes	Yes/weaned by end of RT	Yes/partial recovery	Fatigue, insomnia	6	Dead (25)
2/F	10 years 3 months	4	12	30.6/focal	–	Yes/weaned at progression	No	Vomiting, fatigue, weakness	5	Dead (9)
3/F	5 years	9	14	30.6/focal	Yes	Yes/weaned by end of RT	Yes/fully recovered	None	3	Dead (15)
4/M	13 years^b^	9^c^	24	30.6/whole brain	Yes	Nil	Yes/fully recovered	None	12	Alive (37)
5/F	4 years 9 months^d^	32^c^	75	30.6/whole brain	Yes	Nil	Yes/fully recovered	Fatigue, decreased energy, appetite	7	Dead (45)
6/M	2 years 3 months^e^	13	40	30.6/focal	–	Nil	Yes/fully recovered	None	–	Alive (16)
7/F	5 years	10^f^	60	21.6/focal	Yes	Nil	No	None	–	Dead (7)
8/M	4 years 6 months^g^	6	15	21.6/focal	Yes	Nil	Yes/fully recovered	None	–	Dead (4)
9/F	5 years^h^	36	30	36/focal	Yes	Nil	Yes/partial recovery	None	3	Dead (4)
10/M	9 years^h^	12	30	36/focal	Yes	Yes	Yes/partial recovery	Fatigue	9	Dead (9)

*D, days; Dx, diagnosis; Gy, Gray*.

*^a^This patient had a second relapse 6 months after re-RT and received a third course of re-RT with 21.6 Gy*.

*^b^Started on Temozolomide 3 months after re-RT and continued until progression 1 year after re-RT*.

*^c^Leptomeningeal/parenchymal dissemination at recurrence*.

*^d^Atypical presentation at diagnosis: MRI showed expansive brain stem lesion centered in the pons, and a second non-enhancing lesion in the right cerebellar hemisphere. The lesions were biopsied revealing an anaplastic astrocytoma*.

*^e^Favorable prognostic factors at diagnosis: age <3 years and prolonged interval between onset of symptoms and diagnosis (10 months). Biopsy at diagnosis revealed glioblastoma multiforme*.

*^f^Received Bevacizumab (Avastin), biweekly, at the time of relapse for a total of eight doses*.

*^g^Prolonged interval between onset of symptoms and diagnosis (14 months). The child had an alteration of voice with no other clinical symptoms*.

*^h^Received Valproic acid starting 6 weeks after completion of re-RT*.

### Chemotherapy

Different chemotherapeutic strategies including high-dose myeloablative chemotherapy with stem cell rescue, neo-adjuvant (pre-RT) multi-agent chemotherapy, concurrent chemotherapy with RT (fractionated/hyper-fractionated protocols), and adjuvant chemotherapy have not demonstrated improved survival when compared to RT alone [Tables 3A,B (61–90)]. In particular, the current standard of care for adult GBM, namely RT with concurrent and adjuvant temozolomide, did not improve outcomes for DIPG [(91, 92), Table 4]. Various studies utilizing both neo-adjuvant and concurrent chemotherapies have shown no improvement in the OS of DIPG patients when compared to standard RT. Two French studies showed improved results using chemotherapy with RT when compared to RT alone. Doz et al. (65) used Carboplatin prior to and concurrently with RT in 38 DIPG patients and reported a median survival of 11 months (compared to 9 months with RT alone). Frappaz et al. (67) used frontline chemotherapy (Tamoxifen/BCNU/Cisplatin alternating with high dose methotrexate/HDMTX) aimed at delaying radiation until time of clinical progression. Median survival increased significantly in patients participating in the protocol compared to historical controls (17 vs. 9 months, *P* = 0.022). However there was prolonged hospitalization and increased infections in the chemotherapy group. The German HIT–GBM protocols assessed a variety of chemotherapeutic strategies in the HIT–GBM protocols (A–D) in the treatment of DIPG and could not demonstrate any survival benefit when compared to RT alone (69, 79, 85, 90). In a single institution study over two decades Massimino et al. (68), evaluated four different regimes in the treatment of DIPG: (1) intensive high-dose courses of chemotherapy (cisplatin/etoposide, cyclophosphamide/vincristine/methotrexate) and a subsequent course of myeloablative thiotepa followed by RT and maintenance chemotherapy; (2) cisplatin/etoposide followed by isotretinoin before, during, and after focal RT; (3) concurrent chemo-radiotherapy (etoposide, cytarabine, ifosfamide, cisplatin, and dactinomycin); and (4) Vinorelbine before, during, and after radiotherapy. The median survival time was reported as 11 months with no survival advantage within protocols compared with RT alone (Tables 3A,B).

**Table 3:** **(A) Summary of clinical trials of neo-adjuvant chemotherapy and RT in DIPG; (B) summary of clinical trials of Chemotherapy (pre-/concurrent/adjuvant) and RT in DIPG**.

Reference	Year/phase	Number of patients (*n*)	Treatment	Outcome	RT and comments
Kretschmar et al. (61) POG-8833	1993/II	32	CDDP/CPM – four cycles followed by RT	MST: 9 months	RT dose: 66/60/1.1
Dunkel et al. (62)	1998/pilot	6	BCNU/Etop/Te followed by ABMT and RT	MST: 11.4 (7.6–17.1)	RT dose: 72–78/72–78/1
Jakacki et al. (63)	1999/II	11	CCNU/P/VCR followed by ABMT and RT	MST: 13 months	RT dose: 50.4–59.4/–/1.8
Jennings et al. (64) CCG-9941	2002/II	Group A = 32, Group B = 31	A = VCR/Car/Etop B = CDDP/CPM/Etop/VCR followed by RT	EFS at 1 year-17 ± 5%, 2 years – 6 ± 3%	RT dose: 72/72/1
Doz et al. (65)	2002/pilot	38	Car followed by Car + RT	MST: 11 months	RT dose: 54/30/1.8 Biopsy in 14 patients (HGG-7, LGG-4)
Grundy et al. (66) UKCCSG/SIOP-CNS9204	2010/II	7	Car/CDDP/CPM/HDMTx/VCR followed by RT	Median TTP: 0.21 years (0.1–0.53) Median OS: 0.3 years (0.25–1.43)	
Frappaz et al. (67), SFOP-BSG-98	2008/II	23	BCNU/CDDP/Hydroxyurea/HDMTx/Tamoxifen followed by RT	MST: 17 months (10–23)	RT dose: 54/2/2 Prolonged hospitalization in treated patients
Massimino et al. (68)	2008/II, Study-II	10	CDDP/CPM/Etop/HDMTx/VCR followed by RT	MST: 13 months OS 1 year –70 ± 14%, 2 years – 10 ± 9%	All patients received adjuvant CCNU/VCR chemotherapy post RT
Wolff et al. (69) (HIT–GBM-D)	2011/II	7	HDMTx (two doses) followed by RT + CDDP/Etop/Ifos/VCR	Median EFS: 0.55	All patients received adjuvant CCNU/VCR/Prednisone chemotherapy post RT
Levin et al. (70)	1984/II	28	CCNU/5FU followed by RT	MST: 11 months	RT dose: 55/31/1.8 Patients received adjuvant hydroxyurea/misonidazole post RT Brain stem gliomas (including DIPG) were included in the study
Wakabayashi et al. (71)	1992/Pilot	16	RT + ACNU/IFN-β	MST: 15.7 months	RT dose: 40–60/27–30/1.5–2 Brain stem gliomas (including DIPG) were included in the study
Bouffet et al. (72)	1997/II	8	BCNU-ABMT followed by RT	MST: 4 months	Patients were previously treated with Chemo/RT Brain stem gliomas (including DIPG) were included in the study
Walter et al. (73)	1998/I–II	9	RT + Car/Etop	Median TTP: 8 months OS: 1 year – 44%, 2 years – 11%	RT dose: 70.2/60/1.7
Allen et al. (74)	1999/I–II	31	RT + Car	Median OS: 12 months	RT dose: 72/72/1 Three long-term survivors (mean F/U 79 months)
Jakacki et al. (63)	1999/pilot-II	6	CCNU/P/VCR + ABMT (pre-/during/post) RT	MST: 11 months	RT dose: 50.4–59.4/28–33/1.8
Mandell LR et al. (75)	1999/III (randomized)	Group-I = 66, Group-II = 64	RT + CDDP	Median TTP: 6 months (I)/5 months (II) OS 1 year –30.9(I)/27(II), 2 years – 7.1 (I)/6.7(II)	RT dose: Group-I: 54/30/1.8. Group-II: 70.2/60/1.17 This study showed that hyperfractionation has no benefit compared to routine fractionation
Freeman et al. (76), POG-9239	2000/II	64	RT with and without CDDP	1 year survival lower in CDDP group compared to RT alone	RT dose: 70.2/60/1.7 Brain stem gliomas (including DIPG) were included in the study
Bouffet et al. (77)	2000/Pilot	36	RT followed by Bu/Te and ABMT	MST: 10 months	RT dose: 54/30/1.8 Brain stem gliomas (including DIPG) were included in the study
Broniscer et al. (78)	2000/II	27	RT + tamoxifen followed by Tamoxifen	OS 1 year-37 ± 9.5%	RT dose: 54–60/34–36/1.5–1.8
Wolff et al. (79), HIT–GBM-A	2002/pilot	20	RT + trophosphamide/Etop	OS 1 year-0.4, 4 years-0.05	RT dose: 54/30/1.8 Biopsy in 15 patients (Grade II-4, III-3, IV-8)
Marcus et al. (80)	2003/I	18	RT + Etanidazole	MST: 8.5 months (3–58)	RT dose: 66/44/1.5
Sanghavi et al. (81)	2003/I	16	RT + Topotecan	MST: 15 months (9.6–19) OS 1 year 53%	RT dose: 59.4/33/1.8
Bernier-Chastagnier et al. (82)	2005/II	32	RT + Topotecan	MST: 8.3 months OS 1 year 25.5 ± 8%	RT dose: 54/30/1.8
Packer et al. (83)	2005/I	13	Car + RMP-7 followed by RT	MST: 11 months	RT dose: 59.4/33/1.8 RMP-7 (cereport) is a synthetic bradykinin analog that increases BBB permeability
Greenberg et al. (84)	2005/I	7	RT + Etop/VCR/cyclosporine-A	MST: 11 months	RT dose: 54/30/1.8 Cyclosporine was used for P-gp inhibition
Wolff et al. (85), HIT–GBM-B	2006/I-II	19	RT + CDDP/Etop/Ifos followed by CPM/IFN-γ	MST: 9.6 months	RT dose: 54/30/1.8
Warren et al. (86)	2006/II	12	RMP-7 + Car	No response in any patient	Brain stem gliomas (including DIPG) were included in the study
Turner et al. (87)	2007/II	12	RT + Thalidomide followed by Thalidomide	Median TTP: 5 months (2–11) Median TTD: 9 months (5–17)	RT dose: 55.8/31/1.8
Korones et al. (88)	2008/II	30	RT + VCR/Etop (oral) followed by VCR/Etop (oral) × 10cycles	MST: 9 months (3–36), OS 1 year – 27 ± 7%, 2 year –3 ± 2%	RT dose: 54/30/1.8
Massimino et al. (68)	2008/II, study-I Study-III Study-IV	21 17 14	RT + Ara-C/Act-D/CDDP/Etop/Ifos RT + CDDP Etop/isotretinoin (prior/during and after RT) RT + vinorelbine (prior/during and after RT)	MST: 12 months, OS 1 year – 45 ± 6%, 2 year – 18 ± 5% MST: 9 months, OS 1 year – 29 ± 11%, 2 year –12 ± 8% MST: 9 months, OS 1 year – 43 ± 13%, 2 year – 21 ± 11%	RT dose: 54/30/1.8 (*n* = 18), 66/60/1.1 (*n* = 3) RT dose: 54–60/27–30/1.8–2 RT dose: 54–60/27–30/2
Michalski et al. (89)	2009/II	31	RT + tamoxifen (oral)	MST: 6.32 months, OS 1 year –16.1%	RT dose: 54/30/1.8
Wolff et al. (90), HIT–GBM-C	2010/II	37	RT + CDDP/Etop/VCR followed by CDDP/Etop/Ifos/valproic acid	Median OS: 1.13 (0.87–1.39)	RT dose: 54/30/1.8

*OS, overall survival; PFS, progression free survival; MST, median survival time; TTP, time to progression; TTD, time to death; EFS, event free survival; RT, radiation therapy; RT dose, cumulative dose (Gy)/number of fractions/dose per fraction (Gy); Ara-C, cytosine arabinoside; Act-D, actinomycin D; ACNU, Amino-chloroethyl-nitrosourea hydrochloride; BCNU, Carmustine; Bu, Busulphan; Car, Carboplatin; CCNU, lomustine, CDDP, cisplatin, CPM, cyclophosphamide, Etop, etoposide, 5FU-5 Flurouracil; HDMTx, high dose Methotrexate, Ifos, ifosfamide, P, procarbizine, IFN-β, interferon beta; IFN-γ, interferon gamma; Te, thiotepa, ABMT, autologous bone marrow transplant*.

**Table 4:** **Concomitant and Adjuvant Temozolomide (TMZ) based chemotherapy in DIPG**.

Reference	Year/phase	Number of patients (*N*)	TMZ dose (mg/m^2^) with RT/after RT/number of cycles	Outcome	Comments
Lashford et al. (93)	2002/II	21	–/200/–	MST: 6.2 months	All patients received RT TMZ started at relapse
Broniscer et al. (94)	2005/II	33	–/200/6	MST: 12 months OS: 1 year – 48%	All patients received irinotecan and RT RT dosing: 50.4–70.8/30/1.8
Sirachainan et al. (95)	2008/II	12	75/200/–	Median TTP: 10.2 ± 3 months MST: 13.5 ± 3.6 months OS: 1 year – 58 ± 4.2%	All patients received cis-retinoic acid. RT dose: 35.8-59.4 Gy
Jalali et al. (96)	2010/II	20	75/200/12	Median OS: 9.15 months Median PFS: 6.9 months	RT dose: 54/30/1.8
Chiang et al. (97)	2010/II	I = 10 II = 8	75/150/–	MST: 12.3 months OS: 1 year – 51%	I = adjuvant TMZ II = Concurrent TMZ
Kim et al. (98)	2010/I-II	12	75/200/–	MST: 12.7 months OS: 1 year – 58%	All patients received thalidomide with RT
Sharp et al. (99)	2010/II	15	85/85/–	MST: 9.8 months OS: 1 year – 20%	RT dose: 54/30/1.8
Cohen et al. (91) ACNS0126	2011/II	63	90/200/10	MST: 9 months OS: 1 year – 40%, 2 year – 3.6%	RT dose: 59.4/33/1.8
Chassot et al. (92)	2012/II	21	75/200/6	MST: 11.7 months OS: 1 year – 50%	RT dose: 54/30/1.8 All cases were biopsied
Kebudi et al. (100)	2012/retrospective	21	75/200/12	MST: 12 months OS: 1 year – 55%, 2 year – 37%	
Bailey et al. (101)	2013/II	43	75/75–100/12	MST: 9.5 months OS: 1 year – 35%, 2 year – 17%	
Zaky et al. (102)	2013/retrospective	6	75/200/–	Median EFS: 6.1 months. Median OS: 9.6 months	Patients received concurrent chemo (carboplatin/etoposide) followed by adjuvant chemo (irinotecan/bevacizumab)
Aguilera et al. (103)	2013/report	2	–/200/–	PFS: 37/47 months	RT dose: 54/30/1.8 Both patients received adjuvant bevacizumab and were alive at the time of reporting Tumor size decreased by >65%
Muller et al. (42)	2014/II	2	75/–/–	MST: 11.9/8.1 months	Both patients received nimotuzumab and cranio-spinal RT
Rizzo et al. (104)	2015/II	14	75/200/12	Median TTP: 7.15 months	

*OS, overall survival; PFS, progression free survival; MST, median survival time; TTP, time to progression; EFS, event free survival; RT, radiation therapy; RT dose, cumulative dose (Gy)/number of fractions/dose per fraction (Gy)*.

Various clinical trials using Temozolomide (TMZ) in combination with concurrent RT followed by adjuvant TMZ have shown variable results (91–104) (Table 4). Kebudi et al. (100) in their retrospective analysis of 50 DIPG patients treated with RT and TMZ with and without other chemotherapy showed significantly higher OS in the TMZ treated group. Patients receiving RT + TMZ + chemotherapy (other than TMZ) showed prolonged survival when compared to patients receiving only RT (*P* = 0.005). However a number of other studies (Table 4) have reported no distinct survival advantage of RT + TMZ when compared to RT alone. The HIT-HGG-2007 study is a non-inferiority clinical trial designed to prove the hypothesis that ambulatory TMZ-chemoRT is as effective, but less toxic than the previous HIT–GBM (C–D) trials (42). At the present time, adjuvant chemotherapy is not recommended for DIPG outside of a clinical trial.

### Impediments to Chemotherapy/Targeted Therapy

One of the major limitations of DIPG treatment is the successful and efficient delivery of effective therapies to the tumor. For a drug or a targeted therapeutic agent to be effective, the active compound: (a) should reach its target in the tumor cells, (b) in adequate concentrations/bind to the target present in the tumor cells, (c) for an adequate amount of time for tumor cytotoxicity, and (d) the tumor cells themselves should be sensitive to the drug/agent. There are several factors that can affect drug concentrations within the DIPG tumor (105). These include bio-availability of the drug/agent (serum levels, protein/tissue binding), rate of blood flow to the tumor, degree of brain penetration [blood–brain barrier (BBB) and blood tumor barrier] and drug metabolism. The BBB is frequently intact in DIPG and plays an active role in restricting the delivery of systemically administered conventional and biological therapies (106). This often leads to decreased effective concentration of the therapeutic agents in the tumor.

In order to overcome the restrictive ability of the BBB, several alternative drug delivery strategies have been tried including: transient osmotic disruption of the BBB, enhancing the lipophilicity of drugs, inhibition of membrane efflux pumps, intra-arterial/intra-thecal chemotherapy, intra-nasal chemotherapy, direct intra-tumoral chemotherapy, etc., but each has met with minimal success (107). The most clinically proven approach to increase drug penetration into the CNS is to reversibly disrupt the junctions formed by the endothelial cells to enhance their penetration through intercellular junctions. This can be accomplished through the use of osmotic agents or pharmacologically through the targeting of membrane receptors that alter BBB permeability. While osmotic agents such as mannitol have been used to modulate BBB permeability in both pre-clinical and clinical settings (107) the major drawback with osmotic disruption is the long recovery period required for reestablishment of BBB integrity (108). With several hours required for the return of normal BBB function, patients are susceptible to toxicity (109). On the other hand, pharmacological agents such as RMP-7/Cereport, a bradykinin receptor agonist, have been shown to transiently disrupt the BBB in various animal models (108, 110). However, these agents have failed to produce the desired response in clinical trials due to non-uniform disruption of the BBB (83, 86, 110). Veringa et al. (106) using patient derived cell cultures and tumors showed that DIPG cells express MRP1 and the tumor vasculature exhibits drug efflux transporters (P-gp, MRP1, and BCRP1). However, cell cultures were sensitive to an array of chemotherapeutic agents when treated *in vitro*. The concurrent use of efflux inhibitors (111) along with chemotherapy might improve the efficacy of chemotherapy and could be considered in a prospective clinical trial. Convection-enhanced delivery (CED), using external or implantable subcutaneous pumps, allows intra-tumoral injection of novel therapeutic agents (chemotherapy, cytotoxic interleukins, radio-immunotherapeutic agents), and there are ongoing phase I/II clinical trials in DIPG.

### Biological Targeted Therapy

Due to the availability of tumor tissue (from both autopsy and surgical biopsy samples) in several centers across the world, novel targets have been identified in DIPG and there are several ongoing clinical trials using targeted therapies. The reader is referred to excellent reviews summarizing the past and ongoing clinical trials involving targeted therapies in DIPG (112–114). However, none of these trials have shown any superior survival benefit when compared to RT alone (Table 5) (115–129).

**Table 5:** **Biological/targeted therapies in DIPG**.

Target	Agent	Newly diagnosed or progressive/relapsed, number of patients (*n*)	Median PFS (months)	PFS at 6 months (%)	Reference
EGFR	Erlotinib	Newly diagnosed with RT	8	90	Geoerger et al. (115)
	Gefitinib	Newly diagnosed with RT (*n* = 43)	7.4	88	Pollack et al. (116)
		Newly diagnosed with RT (*n* = 20)	NR	48	Geyer et al. (117)
	Nimotuzumab	Newly diagnosed with RT (*n* = 41)	5.5	NR	Massimino et al. (118)
		Progressive/relapsed (*n* = 44)	1.7	NR	Bartels et al. (119)
EGFR/VEGFR	Vandetanib	Newly diagnosed with RT (*n* = 21)	NR	88	Broniscer et al. (120)
PDGFRA	Imatinib	Newly diagnosed with RT	NR	70	Pollack et al. (121)
VEGF	Bevacizumab	Progressive/relapsed (*n* = 31) Progressive/relapsed (*n* = 2) Newly diagnosed with RT (*n* = 14)	2.3 2.25 8.8	9.7 NR NR	Gururangan et al. (122) Narayana et al. (123) Salloum et al. (124)
mTOR	Temsirolimus	Progressive/relapsed (*n* = 5)	2.5	NR	Geoerger et al. (125)
Farnesyl transferase	Tipifarnib	Newly diagnosed with RT (*n* = 40)	NR	44	Haas-Kogan et al. (126)
Integrins (αvβ3 and αvβ5)	Cilengitide	Progressive/relapsed (*n* = 31)	NR	NR	MacDonald et al. (127)
Histone de-acetylase (HDAC)	Valproic acid Panabinostat	Newly diagnosed with RT/chemotherapy Murine DIPG models	9.5 –	NR –	Felix et al. (128) Grasso et al. (129)

*PFS, progression free survival; RT, radiation therapy; NR, not recorded*.

### Immunological/Vaccine Therapy

Immunotherapy in DIPG is limited due to the non-availability of tumor tissue to identify and generate tumor-associated antigens. Li et al. (130) evaluated the expression of Epidermal Growth Factor Receptor variant III (EGFRvIII) in postmortem DIPG tissue and primary cell cultures using various laboratory techniques (immunohistochemistry/IHC, western blot, RT-PCR, and flow cytometry). IHC revealed expression of EGFRvIII in four of nine cases and the staining pattern was similar to the adult tumors. Overall expression was detected in 6 of 11 cases. There are several ongoing phase III clinical trials using a peptide vaccine targeting EGFRvIII in the treatment of newly diagnosed GBM in adults. Zhou et al. (131) evaluated the expression of B7-H3, a membrane protein (CD276) against which a therapeutic monoclonal antibody is available (8H9). IHC revealed B7-H3 immunoreactivity in 100% (*n* = 9/9) of postmortem DIPG samples.

Pollack et al. (132) conducted a pilot study using a peptide-based vaccine against novel GAA [Glioma associated antigens – EphA2, interleukin-13 receptor alpha2 (IL-13Rα2), and survivin]-derived epitopes in children with DIPG and HGGs. Newly diagnosed DIPG patients [treated with RT alone (*N* = 14) and RT + chemotherapy (*N* = 8)] were administered the vaccine in conjunction with immuno-adjuvant polyinosinic-polycytidylic acid (poly [I: C]) stabilized by lysine and carboxymethylcellulose (poly-ICLC). Five children had symptomatic pseudo-progression, which responded to dexamethasone and was associated with prolonged survival. The vaccine was well tolerated overall with good clinical (OS between 6.3 to >38 months) and immunological response (positive anti-GAA immune response in 8 patients). Based upon the feasibility, safety, and potential promise of these studies, immune-based therapeutic strategies are expected to be offered in prospective phase II and III clinical trials.

## Prognostic/Biologic Markers in DIPG

Although the outcome of DIPG is extremely poor and uniformly fatal, there are a sub-group of patients who survive longer. Some of the favorable clinical prognostic factors are young age at diagnosis (<3 years), prolonged interval between onset of symptoms and diagnosis (>6 months) and absence of cranial nerve palsies or long tract involvement at presentation. Tools to assess the efficacy of treatment and monitoring in DIPG are evolving. The RANO (Response Assessment in Neuro-oncology) criteria (using MRI) are used to assess response during or post chemotherapy/targeted therapy with decrease in steroid use, tumor size and improved neurologic symptoms indicative of response to therapy. However, DIPG tumors frequently do not enhance or may exhibit variable patterns of enhancement and either decrease in tumor size is not sustained or this does not translate into improved survival (133). Moreover, conventional MRI instructs us regarding tumor structure and location and cannot reliably differentiate therapy-related phenomena such as efficacy, pseudo-progression, or pseudo-response (133). Hipp et al. (134) used prospective multi-parametric imaging to evaluate outcome in children with DIPG. In their study, increased perfusion [using dynamic susceptibility contrast (DSC) MRI] at any single time point was associated with shorter survival (RR = 4.91), and increasing perfusion over time was a poor prognostic factor. Steffen-Smith et al. (135) used Magnetic Resonance Spectroscopy (MRS) techniques to monitor DIPG patients throughout their course of treatment. In their study, the choline: *N*-acetylaspartate ratio (CHO:NAA) was shown to be prognostic; patients with a CHO:NAA ratio higher than the median of 2.1 demonstrated a greater risk of early mortality compared to patients with a ratio of ≤2.1. During follow-up, changes in this ratio had an impact on prognosis with increase in the CHO: NAA ratio being inversely associated with survival and decreasing CHO: NAA ratio being associated with a longer life expectancy. Although very promising, these imaging modalities are limited by their availability in specialized centers and only as part of research protocols.

Diffuse intrinsic pontine glioma patients who have histone mutations (H3.3 and H3.1, K27M) identified by IHC and other methods have a worse OS when compared to wild-type tumors, and this association is independent of patient age and histological diagnosis. Jansen et al. (136) recently developed a DIPG survival prediction tool that can be used for predicting outcome and risk stratification for prospective future clinical trials. Multivariate Cox proportional hazards analysis was used to pick both positive prognostic predictors (longer symptom duration, age ≤3 years and use of concurrent oral and intravenous chemotherapy with RT) and negative predictor (presence of ring enhancement in contrast MRI). The risk score calculated based on these predictors will not only able to predict the prognosis but also stratify patients into risk groups (standard, intermediate, and high), which can be used in future clinical trials. This prediction model itself requires validation in a prospective clinical trial.

## Summary

In summary, diagnostic and therapeutic failures of the past and recent advances in the genomics and biology of DIPG have made us wiser in the following ways: (1) we now understand that DIPG is a heterogeneous group of tumors with origins that can be traced to aberrant neurodevelopment and it is biologically distinct from other pediatric and adult HGG. Therefore, adapting chemotherapy and/or targeted therapy used in pediatric and adult HGG for DIPG should be abandoned. (2) biopsy of DIPG is relatively safe using modern neurosurgical techniques and should be considered in the context of clinical trials. Biopsy results will confirm the diagnosis, may identify actionable targets, and with the use of appropriate prediction tools, help us to risk-stratify these patients for both therapy and prognosis. (3) there is evidence in the literature to suggest that DIPG is a whole brain disease and this may support whole neuraxis imaging at diagnosis, during therapy and at follow-up. (4) previous decades of treatment failure may be attributed to several factors, including: inadequate drug levels within the tumor due to lack of BBB permeability, single agent testing, signaling pathway redundancy leading to drug resistance, lack of patient pre-selection, lack of biomarkers to assess response and off-target effects. In the future, judicious use of radiation may also include appropriate concurrent/adjuvant therapies directed against signaling pathways, genetic or epigenetic targets; BBB permeability agents and/or anti-invasive/anti-migration agents might improve the efficacy of these combined treatment modalities. (5) Pre-clinical testing and validation of appropriately chosen targets [e.g., JMJD3, H3K27 demethylase (137), Menin (138), ACVR1 (24)] in available tumor models (138, 139) will help to identify novel effective therapies and provide options to personalize treatment and limit side effects and long-term toxicities. Expedited clinical trials using these targets in small numbers of patients in tertiary centers will facilitate the development of larger multicenter randomized clinical trials. Use of validated biomarkers (140) along with appropriate neuraxis (brain and spine) and advanced imaging techniques (DSC–MRI, MRS) could assist us to monitor patients with DIPG for therapy response and toward earlier detection of relapse/progression. The time has come for the pediatric neuro-oncology community to unite in their efforts through the rapid integration of data from basic science and translational research along with appropriately conducted clinical trials to conquer this deadly disease.

## Conflict of Interest Statement

The authors declare that the research was conducted in the absence of any commercial or financial relationships that could be construed as a potential conflict of interest.
